# *Htr1b* is necessary for normal retinal function in mice

**DOI:** 10.3389/fncel.2025.1690447

**Published:** 2025-11-17

**Authors:** Solomon E. Gibson, Xiaofeng Tao, Guofu Shen, Justin Ma, Yong H. Park, Maria Polo-Prieto, Benjamin J. Frankfort

**Affiliations:** 1 Department of Ophthalmology, Baylor College of Medicine, Houston, TX, United States; 2 Department of Neuroscience, Baylor College of Medicine, Houston, TX, United States

**Keywords:** retina, serotonin, 5-HT, 5-hydroxytryptamine, serotonin receptor 1B (HTR1B), 5-HT1B, retinal ganglion cell (RGC)

## Abstract

**Introduction:**

Serotonin (5-HT) is a neurotransmitter that is involved in retinal development, physiology, and vision, yet the specific contribution of individual 5-HT receptors to retinal function is poorly characterized. We identified 5-HT receptor 1B (*Htr1b*) as a potential key regulator of serotonergic signaling in the retina.

**Methods:**

*Htr1b* localization was examined using RNAseq and *in situ* labeling. Retinal structure was assessed using histology and SD-OCT. Visual function was evaluated using optomotor behavioral experiments. Retinal function was characterized *in vivo* using electroretinography (ERG) and *ex vivo* using multielectrode array (MEA) recordings.

**Results:**

*Htr1b* transcript and HTR1B protein localized primarily to the inner retina and RGCs. While *Htr1b*^–/–^ mice displayed normal retinal anatomy, they exhibited visual deficits in contrast sensitivity and visual acuity. ERG recordings revealed that RGCs had latency delays and reduced sensitivity to changes in light intensity. MEA analysis showed altered RGC firing patterns and increased variability following 5-HT application. These effects were cell-type specific: *Htr1b*^–/–^ ON RGCs showed elevated basal firing rates while *Htr1b*^–/–^ OFF RGCs showed reduced 5-HT responses.

**Discussion:**

These findings demonstrate that *Htr1b* is necessary for normal retinal serotonergic signaling and contributes to the regulation of RGC excitability and visual sensitivity.

## 1 Introduction

Serotonin, also known as 5-hydroxytryptamine (5-HT), is an important modulatory neurotransmitter with broad relevance to nervous system development and behavioral, cognitive, and physiological function (Jacobs and Azmitia, 1992; Mosienko et al., 2015). 5-HT acts by binding to 5-HT receptors (HTRs), which are grouped into 7 families based on their function and genetic similarity (Hannon and Hoyer, 2008; Wirth et al., 2017). 5-HT receptors have a wide range of effects based on their position in the synapse which includes pre-synaptic autoreceptors where they regulate 5-HT release/reuptake and post-synaptic heteroreceptors where they regulate 5-HT effects on a recipient cell (Nautiyal et al., 2016; Li et al., 2022). Both can impact either excitatory or inhibitory processes through direct action via G-protein coupled receptors or through indirect modification of a wide variety of signaling pathways (Boess and Martin, 1994; Sari, 2004; Hannon and Hoyer, 2008).

In the retina, 5-HT is involved in a wide variety of processes. 5-HT impacts neural proliferation and development by regulating morphogenesis (De Lucchini et al., 2005; George et al., 2005; Bonnin et al., 2007), neurite outgrowth (Trakhtenberg et al., 2017), and cell regeneration (Sobrido-Cameán et al., 2018). 5-HT modulates visual processing by regulating responses to light and looming stimuli and retinal information flow to the thalamus (Thier and Wässle, 1984; Brunken and Jin, 1993; Reggiani et al., 2023). In retinal ganglion cells (RGCs), 5-HT can directly impact firing rate, ON-OFF properties, axon guidance, and neurite extension (Brunken and Daw, 1988b; Jin and Brunken, 1998; Trakhtenberg et al., 2017; Zhou et al., 2019a,b). 5-HT may also play a neuroprotective role in retinal pathology, as pharmacological activation or blockade of 5-HT receptors is protective against several retinal diseases including myopia, retinal ischemia, light-induced retinopathy, and glaucoma (Inoue-Matsuhisa et al., 2003; George et al., 2005; Collier et al., 2011; Tullis et al., 2015; Zhou et al., 2019b). Furthermore, oral intake of selective serotonin reuptake inhibitors (SSRIs) has been associated with positive outcomes in diseases such as glaucoma, macular degeneration, and diabetic retinopathy (Yekta et al., 2015; Zheng et al., 2018; Ambati et al., 2021).

Despite its importance in vision, 5-HT circuitry in the retina remains poorly defined. A17 amacrine cells, bipolar cells, and RGCs are able to accumulate 5-HT (Kolb, 1995; Menger and Wässle, 2000; Ghai et al., 2009), Additional evidence suggests that retinopetal serotonergic projections from the dorsal raphe may supply 5-HT to the retina in some species (Lima and Urbina, 1998; Gastinger et al., 2006). However, the location of 5-HT synthesis has not been established in the mouse (Yan et al., 2020).

Regardless of the source of 5-HT, many 5-HT receptors are present in wide distributions throughout many cell types in the retina, and little is known about their organ-specific functions (Sharif and Senchyna, 2006; Fadl et al., 2020). By systematically studying these receptors, we can identify the mechanisms that underlie serotonin-related responses in the retina.

To begin our investigation of 5-HT receptors in RGCs, we re-analyzed several RNA sequencing databases to determine the expression of all *Htr* genes. We found that *Htr1b* is highly expressed in RGCs, and especially αRGCs, and this expression is conserved in human retinas. Concomitant loss of function studies in mice reveal that *Htr1b* is necessary for normal vision, retinal electrophysiology, RGC firing rates, and response to 5-HT.

## 2 Methods and materials

### 2.1 Animals

*Htr1b*^–/–^ mice (JAX 029609) were obtained on a BALB/cJ strain and then backcrossed to C57BL/6J for 5 generations prior to experimentation. This *Htr1b* mutation is a functional null in which a neo-cassette was inserted into the coding region of the single exon *Htr1b* gene (Saudou et al., 1994). We confirmed the presence of the JB allele using Sanger sequencing in which we targeted and amplified the mutant *Htr1b* strand (5′-CTTCTATCGCCTTCTTGACG). All experiments were conducted on 8–10 weeks old mice both male and female mice were used in approximately equal numbers. Animal procedures were approved by the Baylor College of Medicine IACUC and performed per NIH guidelines and the ARVO Statement for the Use of Animals in Ophthalmic and Vision Research.

### 2.2 *Htr* gene expression

To obtain gene expression data for adult mouse RGCs, we analyzed a previously published bulk RNA sequencing dataset GSE122205 (Park et al., 2019). We analyzed the sequence data using bowtie2 (v2.2.3), cufflinks (v2.2.1), and cuffdiff pipeline to determine the relative expression level of all known *Htr* genes. In addition, we obtained single-cell RNA-sequencing (scRNA-seq) databases derived from both mouse GSE153674, GSE137400 (Tran et al., 2019; Fadl et al., 2020) and human E-MTAB-7316 (Lukowski et al., 2019) retinas. We re-analyzed the scRNA-seq data using the Seurat package in R4 (Hao et al., 2021).

### 2.3 Antibody staining

Mice were euthanized via cervical dislocation while under isoflurane-induced unconsciousness, followed by decapitation. Retinas were then explanted and prepared as previously described (Shen et al., 2018) and stained with primary antibodies to serotonin receptor 1B (HTR1B; 1:250, Abcam, Cambridge MA), RNA binding protein with multiple splicing (RBPMS; 1:250, PhosphoSolutions, Aurora CO), and class III beta-tubulin (TUJ1; 1:500, Covance, Princeton, NJ, USA). DAPI (1:1000, Abcam. Cambridge MA) was used for nuclear counterstaining. Visualization was achieved using appropriate secondary antibodies and nuclei were counterstained with a fluorescent dye (To-Pro-3; 1:1000, Molecular Probes, Eugene OR). Images were acquired using a laser confocal microscope (LSM 800; Carl Zeiss, Oberkochen, Germany).

### 2.4 *In situ* hybridization

*In situ* hybridization (ISH) was performed using RNAscope technology (ACD Bio, Newark CA) in accordance with the manufacturer’s instructions. To detect *Htr1b*, probes (REF: 312301) were applied to 20 μm retinal cryosections, followed by DAPI staining.

### 2.5 Image analysis

To determine RGC counts, retinas were visualized using a 20x objective on a laser confocal microscope at eight sites across the retinal surface - four near the optic nerve head (central) and four at the retinal periphery - in accordance with an established protocol (Frankfort et al., 2013). The identity of these regions was masked, and RBPMS-positive cells were semi-automatically counted using ImageJ software (National Institutes of Health, Bethesda, MD) from all regions. The total cell density was found by dividing the cell count by the image size.

### 2.6 Optokinetic responses

Bilateral measurements of optokinetic responses (OKRs) were taken using a custom, established, OKR-based technique in conjunction with a commercial system. All behavioral experiments were conducted between the hours of 12–4pm to minimize the variability due to circadian fluctuations in 5-HT. In all cases, the measurements were conducted by the same trained observer who was unaware of the genotypes of the animals. Observers tracked reflexive head-tracking movements in response to a rotating grating stimulus, and a two-alternative-forced-choice (2AFC) paradigm was used to confirm the correct responses. For the custom system, following a minimum of 2 h of dark adaptation, scotopic and photopic contrast sensitivities were measured as previously described (van der Heijden et al., 2016). Contrast sensitivity options ranged from 0 to 100% based on a maximum and minimum screen brightness of 2.3 and −0.8 log cd/m^2^ respectively, at a constant spatial frequency of.081 cycles/degree and speed setting of 2 Hz. For the commercial system, OptoMetry software and equipment were used to assess the contrast sensitivity function and spatial frequency threshold, in accordance with the manufacturer’s instructions (Cerebral Mechanics, Inc., Medicine Hat, AB, Canada). For visual acuity tests, the contrast sensitivity function was generated over 5 spatial frequency gratings (0.031, 0.064, 0.092, 0.103, and 0.192 cycles/degree), as pre-set by the manufacturer, while speed was set to 12 *^o^*/s.

### 2.7 Electroretinogram recordings

The mice were prepared for electroretinogram (ERG) recording as previously described (Frankfort et al., 2013). Briefly, the mice were dark-adapted for 2 h prior to the experiment, and all procedures were conducted under dim red light. We generated scotopic flashes using cyan light emitting diodes, with a 5 ms stimulus of 500 nm wavelength light used for all flashes. We obtained positive scotopic threshold responses at 3 levels of light intensity ranging from −1.91 to −1.04 log R*/rod (photoisomerizations per rod), and the b-wave was measured at 6 light intensities from 0.20 to 2.87 log R*/rod. During acquisition, the ERGs were bandpass filtered from 0.1 to 1000 Hz (Grass Instruments, West Warwick, RI), and the acquired data were sampled at a rate of 10,000 Hz. Custom software in MATLAB (MathWorks, Natick, MA) was used to analyze the traces. The amplitudes of the b-wave and pSTR were separately plotted against the light intensity, and the slope of each was calculated (Shen et al., 2018).

### 2.8 Multielectrode array recording

Multielectrode array (MEA) recordings were used to detect RGC spiking as described previously (Cowan et al., 2016a; Sabharwal et al., 2017; Tao et al., 2019). Animals were dark-adapted for 2 h before being euthanized. Retinas were promptly dissected in carboxygenated mouse Ringer’s solution at ambient temperature under infrared illumination and transferred to an array (MEA-60; Multichannel System MCS GmbH, Reutlingen, BW, Germany) with the RGC side facing down and touching the array. The array was continuously perfused with oxygenated Ringer’s solution (in mM: 124 NaCl, 2.5 KCl, 2 CaCl_2_, 2 MgCl_2_, 1.25 NaH_2_PO_4_, 26 NaHCO_3_, 22 glucose, pH titrated to 7.35, and bubbled with 95% O_2_ and 5%) heated to 35°C during the entire experiment. RGC spiking signals were collected from 60 electrodes arranged in an 8 × 8 grid covering an area measuring approximately 0.6 mm^2^. The RGC signals were sampled at 20 kHz and filtered through a 0.1 Hz high-pass filter. Spike isolation and sorting procedures were performed offline using custom and boutique MATLAB scripts as described previously (Cowan et al., 2016b; Tao et al., 2020) (MathWorks Inc., Natick, MA, USA).

### 2.9 Light stimulation for MEA recording

Visual stimuli were generated with PsychToolbox (MATLAB) and presented on an OLED microdisplay (eMagin Inc., Hopewell Junction, NY). These images were optically projected onto the RGCs through a beam splitter (Edmund Optics). The recordings were performed at photopic intensity levels with a mean illuminance of 3.07 log (R*/rod/sec). The visual stimulation consisted of 3 repetitions of an ON and OFF light step.

ON-OFF functional classification was determined as previously described (Tao et al., 2019).

### 2.10 Pharmacological exposure

Multielectrode array (MEA) responses were recorded under 2 pharmacological conditions. First, baseline recordings were taken, in which the retina was perfused with normal Ringer’s solution. Then, 100 μM of 5-HT (Sigma-Aldrich, H9523) was added to the solution before another set of recordings. For all experiments, retinas were acclimated to the pharmacological condition for 40 min prior to recording. The entire procedure lasted approximately 2 h per retina.

### 2.11 Statistical analysis

Data were analyzed and figures were generated using GraphPad Prism 9 (GraphPad Software, San Diego, CA). All error bars represent the SEM, and statistical analysis was determined using a student *t*-test or 2-way ANOVA followed by Tukey HSD test where appropriate. A cutoff *p*-value of 0.05 was used in all cases.

## 3 Results

### 3.1 *Htr1b* gene and HTR1B protein expression in the retina

**FIGURE 1:**
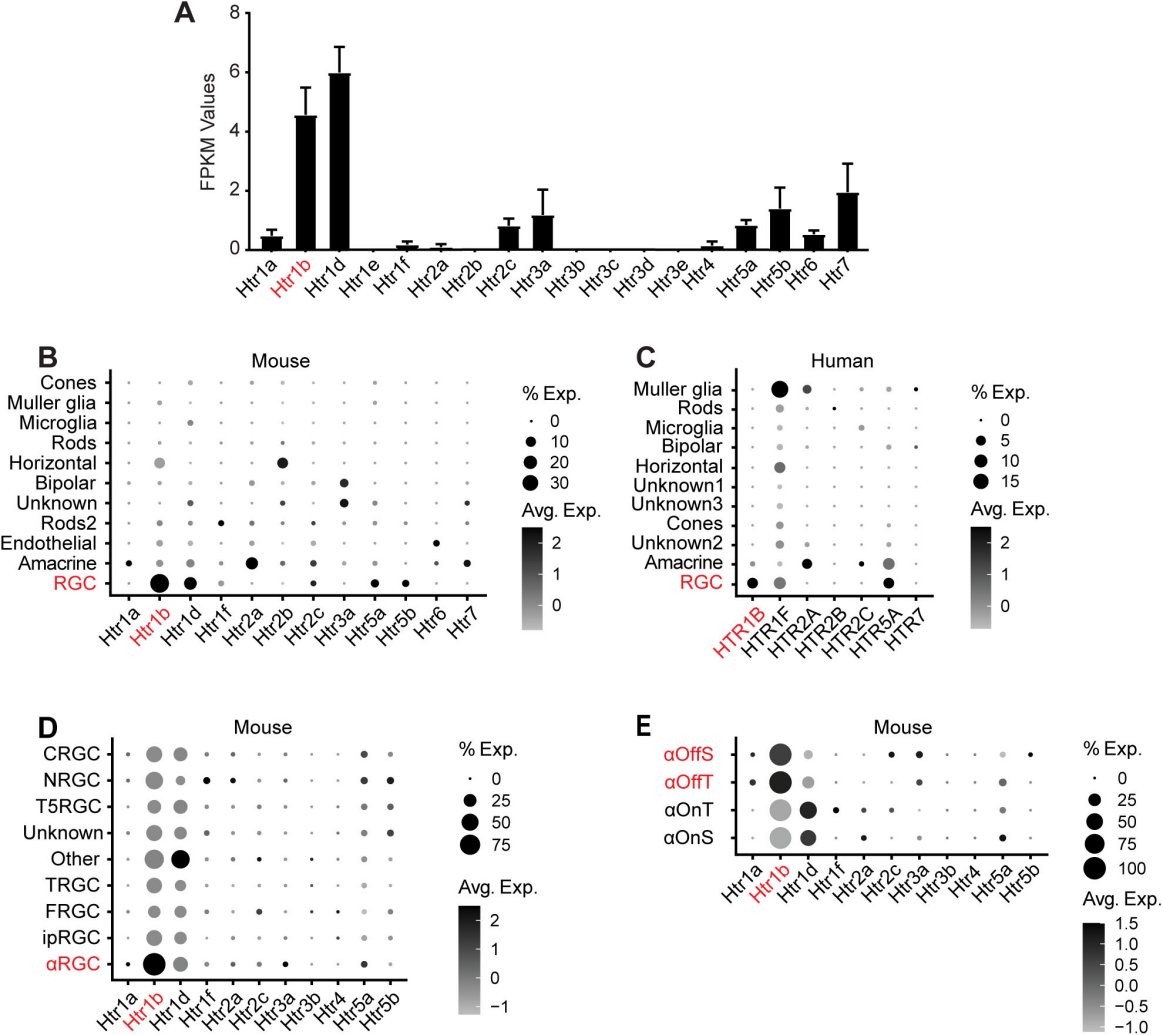
*Htr1b* expression in RGCs. **(A)** Analysis of mouse RGC bulk RNA sequencing data (Park et al., 2019) reveals that *Htr1b* and *Htr1d* are expressed at the highest relative levels among 5-HT receptor transcripts. Fragments per kilobase of transcript per million mapped reads (FPKM) is a normalization method to quantify gene expression levels. **(B)** Single-cell RNA sequencing of all mouse retinal cell types at 8 weeks of age (Fadl et al., 2020) shows that *Htr1b* expression is greatest in RGCs, with much less expression elsewhere in the retina. **(C)** Analysis of a scRNA-seq dataset from human retinal cells (Lukowski et al., 2019) confirms expression of *HTR1B* at high relative levels throughout the retina. HTR1D, despite widespread expression in mouse scRNA-seq datasets, was not expressed in the human scRNA-seq dataset and therefore not included in the figure. **(D)** Analysis of a mouse RGC scRNA-seq dataset reveals 5-HT receptor expression across multiple RGC subtypes, with *Htr1b* expressed most highly in αRGCs. **(E)** Among αRGCs, *Htr1b* is more strongly expressed in OFF αRGCs. “% Exp” refers to the percentage of cells that express a given transcript, and “Avg Exp” refers to the normalized expression level of that transcript.

To identify potential 5-HT receptors in RGCs, we re-analyzed bulk RNA-sequencing data from a published dataset generated from adult, immunopanned, mouse RGCs (Park et al., 2019). We found that twelve *Htr* genes were expressed in RGCs (Figure 1A). *Htr1b* and *Htr1d* were expressed at the highest relative levels, which suggests that these may be the primary *Htr* genes in adult RGCs. To confirm and expand on these findings, we re-analyzed two published scRNA-seq datasets from mouse (Figure 1B) and human (Figure 1C) adult mixed retinal cells to determine the specific expression distribution of HTR genes in RGCs (Lukowski et al., 2019; Fadl et al., 2020). In mice, we confirmed that *Htr1b* is the dominant *Htr* gene in RGCs and is expressed in a high percentage of RGCs (Figure 1B), but not other retinal cell types. Similarly, in humans, we confirmed that *HTR1B* is a major *HTR* gene and that *HTR1D* is not expressed in RGCs, suggesting a potentially conserved function for *HTR1B* but not *HTR1D* between mouse and human (Figure 1C). For this reason, we chose to focus our mouse experiments on *Htr1b*.

Finally, we re-analyzed a third scRNAseq data set to determine which RGC subtypes are most likely to express *Htr1b* (Tran et al., 2019). We found that *Htr1b* is expressed in many RGC subtypes but is most highly expressed in αRGCs (Figure 1D). Furthermore, among αRGC subtypes, *Htr1b* is expressed at highest levels in OFF αRGCs (Figure 1E), highlighting potential subtype specific functions.

**FIGURE 2:**
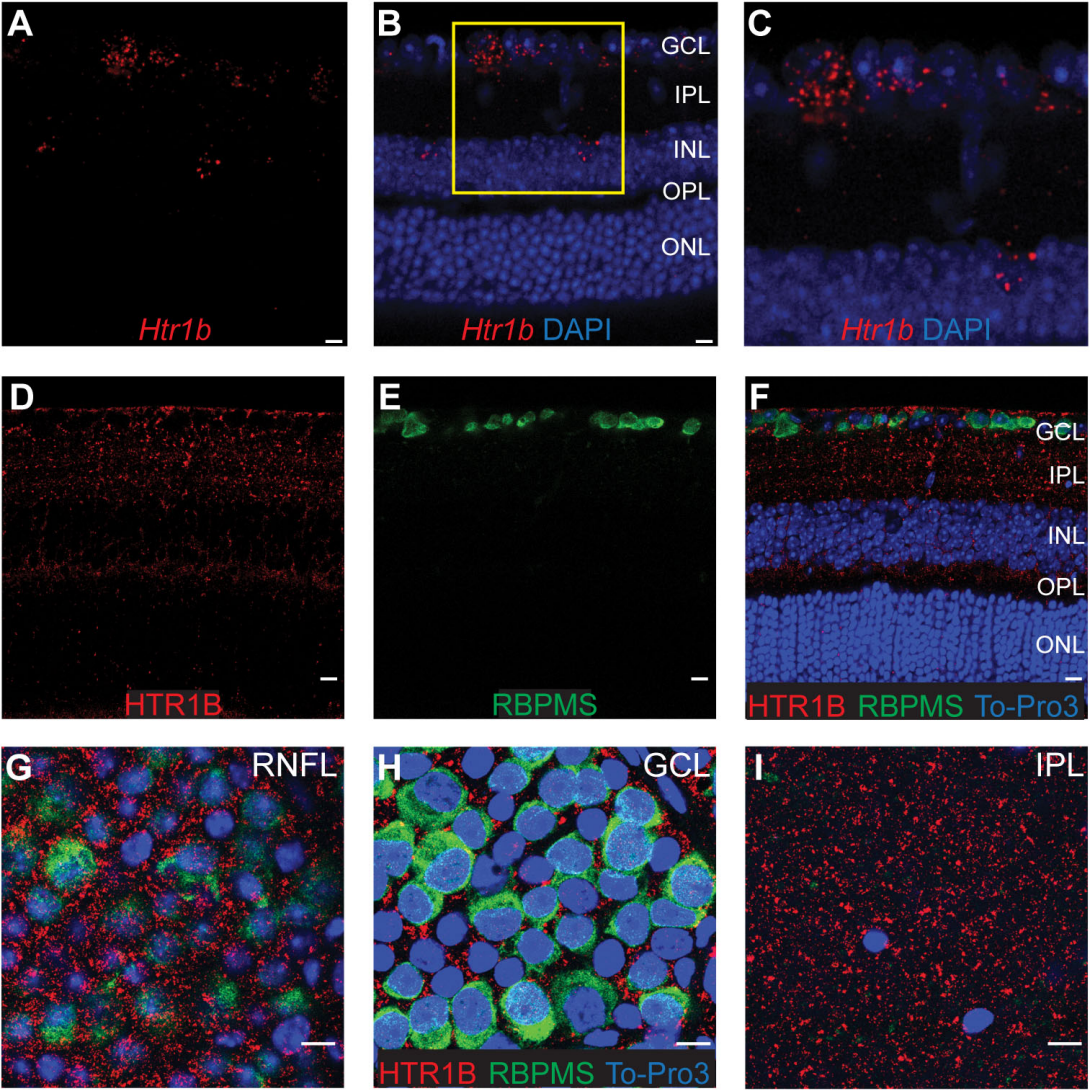
Retinal expression of *Htr1b* and HTR1B. **(A–C)** *In situ* hybridization detects *Htr1b* transcripts (red) in retinal sections. *Htr1b* is most prominent in the ganglion cell layer (GCL). DAPI (blue) labels nuclei. **(C)** Close-up of region **(B)** marked in yellow. Occasional *Htr1b* transcript is seen in the inner nuclear layer (INL). (**D–F)** Immunostaining of retinal sections using antibodies to HTR1B (red) and RBPMS (green) alongside the nuclear dye To-Pro-3 (blue) shows that HTR1B mouse protein expression is highest in the GCL and inner plexiform layer (IPL). Staining in serial sections from the same area of flat mount retinas focused on the retinal nerve fiber layer (RNFL; **G)**, GCL **(H)**, and IPL **(I)** indicates that HTR1B expression is non-nuclear, surrounds the cell membrane of cells in the GCL, and forms puncta in the IPL. Scale bar = 20 μm.

We next investigated the expression pattern of *Htr1b* in the retina by performing fluorescent *in situ* hybridization experiments using probes to detect *Htr1b* mRNA in retinal cross sections (Figures 2A–C). Our results showed that *Htr1b* is expressed primarily in the ganglion cell layer (GCL) with some expression in the inner nuclear layer (INL), consistent with scRNAseq data. We also used antibody staining on retinal sections to localize protein expression (Figures 2D–F). HTR1B puncta were detected throughout the retinal nerve fiber layer (RNFL), GCL, and INL. To further understand the cellular localization of HTR1B, we stained and visualized whole mount retinas using thin Z stack sections (Figures 2G–I) and found that HTR1B expression did not localize to the soma (Figure 2H). Instead, it was predominantly expressed in the RNFL and IPL (Figures 2G, I) and peri-somatically. As expected, these findings suggest that HTR1B is not a somal receptor but instead localizes to the cell surface. While the majority of HTR1B expression was in the inner retina, we also found weaker HTR1B labeling in the OPL, which likely reflects low level receptor expression in a subset of horizontal cells, consistent with scRNA-seq data (Figure 1B).

### 3.2 Gross anatomy of the retina is unaffected in *Htr1b*^–/–^ animals

**FIGURE 3:**
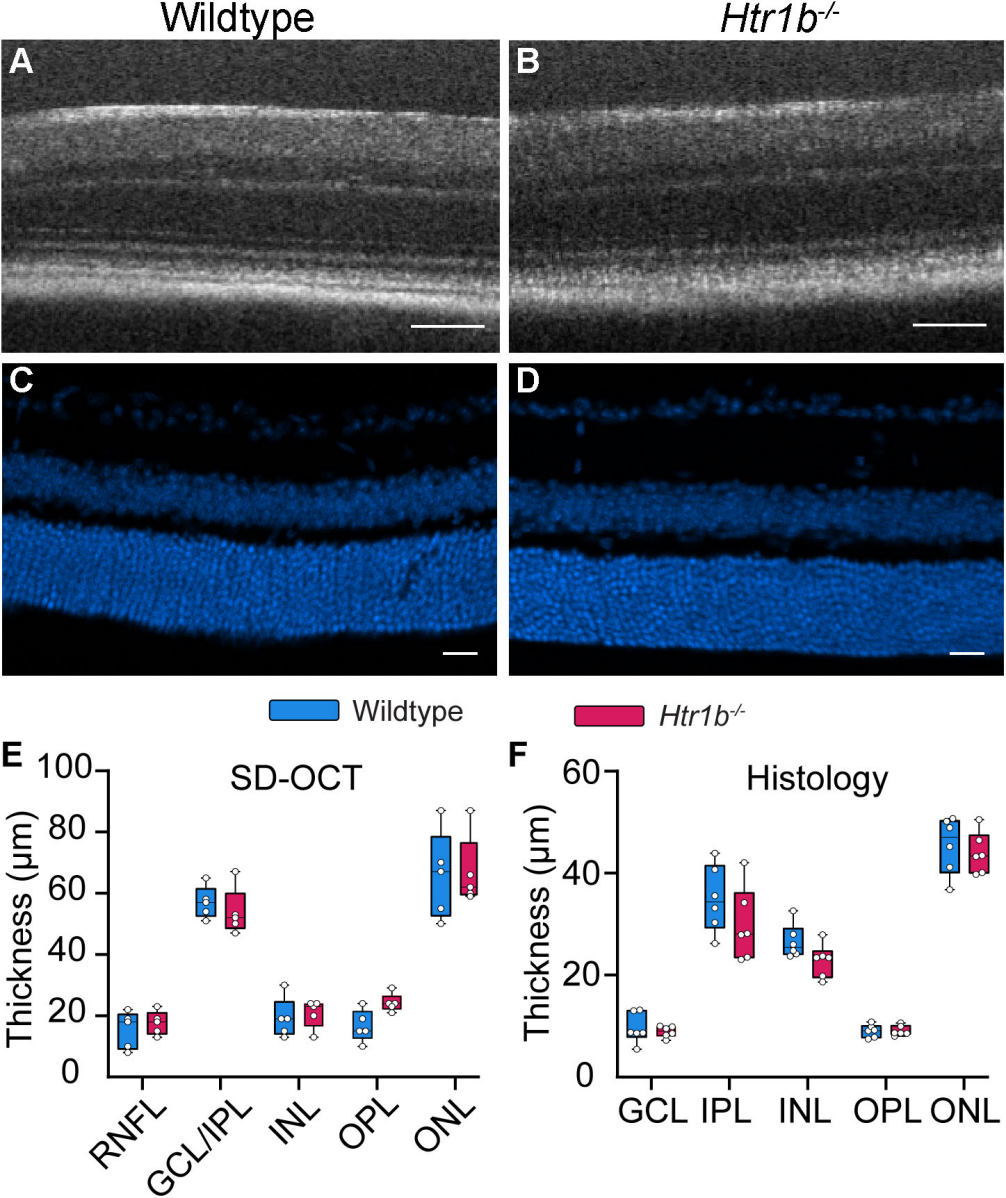
*Htr1b*^–/–^ mice have normal retinal anatomy. **(A,B)** Representative SD-OCT images and **(C,D)** immunohistochemistry sections were prepared from WT and *Htr1b*^–/–^ retinas at 8 weeks of age. DAPI is shown in blue. **(E,F)** The retinal thickness of each layer was manually measured and quantitatively compared. There was no significant difference in retinal thickness at any retinal layer. Scale bar for OCT and IHC: 50 and 20 μm, respectively. For SD-OCT: *Htr1b*^–/–^ *N* = 5 mice; WT *N* = 5 mice. For histology, *Htr1b*^–/–^ *N* = 23 sections across 6 mice; WT *N* = 23 sections across 6 mice.

To evaluate the consequence of *Htr1b* loss of function, we obtained a well-characterized mutant *Htr1b* mouse line in which a neo cassette was inserted into the coding region of the single exon *Htr1b* gene, forming a functional null allele (Saudou et al., 1994). Previous data have shown that genetic manipulation of 5-HT impacts brain development, such that abnormal serotonergic signaling can lead to structural and functional deficits (Bonnin et al., 2007; Daubert and Condron, 2010). Thus, we wanted to evaluate both retinal structure and function in *Htr1b*^–/–^ mice. To assess anatomy, we assessed the thickness of retinal layers *in vivo* using spectral domain optical coherence tomography (SD-OCT) and found no significant difference in thickness at any layer (Figures 3A, B, E and Table 1, *P* > 0.05 for all). We also used histology to measure the thickness of each layer of the retina (Figures 3C, D, F) and, similarly, found no changes in retinal layer thickness in *Htr1b*^–/–^ animals (*P* > 0.05 for all; Table 2). Since *Htr1b* is most highly expressed in RGCs, we extended this analysis to include RGC numbers by assessing RGC somas in the GCL of retinal flat mounts (Table 3). RGC density was analyzed in both central and peripheral regions of the retina, and no significant differences were seen in either region (*P* > 0.05). These findings suggest that *Htr1b* loss of function does not grossly impact the anatomy of the retina or RGC number.

**TABLE 1:** Retinal thickness measurements of SD-OCT images.

	Thickness (mean ± SEM μm)	
Layer	WT	*Htr1b* ^–/–^
RNFL	15.4 ± 2.71	17.6 ± 1.72
GCL/IPL	57 ± 2.35	53.8 ± 3.45
INL	19.2 ± 2.94	20.8 ± 2.08
OPL	16.6 ± 2.34	24.2 ± 1.32
ONL	65.8 ± 6.46	66.8 ± 5.19

Retinal layer thickness for SD-OCT images. *P* > 0.05 for all layers. *N* = 5 retinas per genotype.

**TABLE 2:** Retinal thickness measurements of histology sections.

	Thickness (mean ± SEM μm)	
Layer	WT	*Htr1b* ^–/–^
GCL	9.58 ± 1.21	8.86 ± 0.45
IPL	34.93 ± 2.68	29.79 ± 2.95
INL	26.52 ± 1.37	22.78 ± 1.34
OPL	9.00 ± 0.51	9.03 ± 0.40
ONL	45.45 ± 2.28	43.89 ± 1.66

Retinal layer thickness for histology sections. *P* > 0.05 for all layers. *N* = 6 retinas per genotype.

**TABLE 3:** RGC counts.

	Cell density (mean ± SEM per μm^2^)	
Region	WT	*Htr1b* ^–/–^
Central	3429 ± 222	3920 ± 157
Peripheral	2907 ± 187	2945 ± 217

RGC cell densities. *P* > 0.05 for all comparisons. *N* = 6 retinas per genotype.

### 3.3 *Htr1b*^–/–^ animals have reduced contrast sensitivity and visual acuity

**FIGURE 4:**
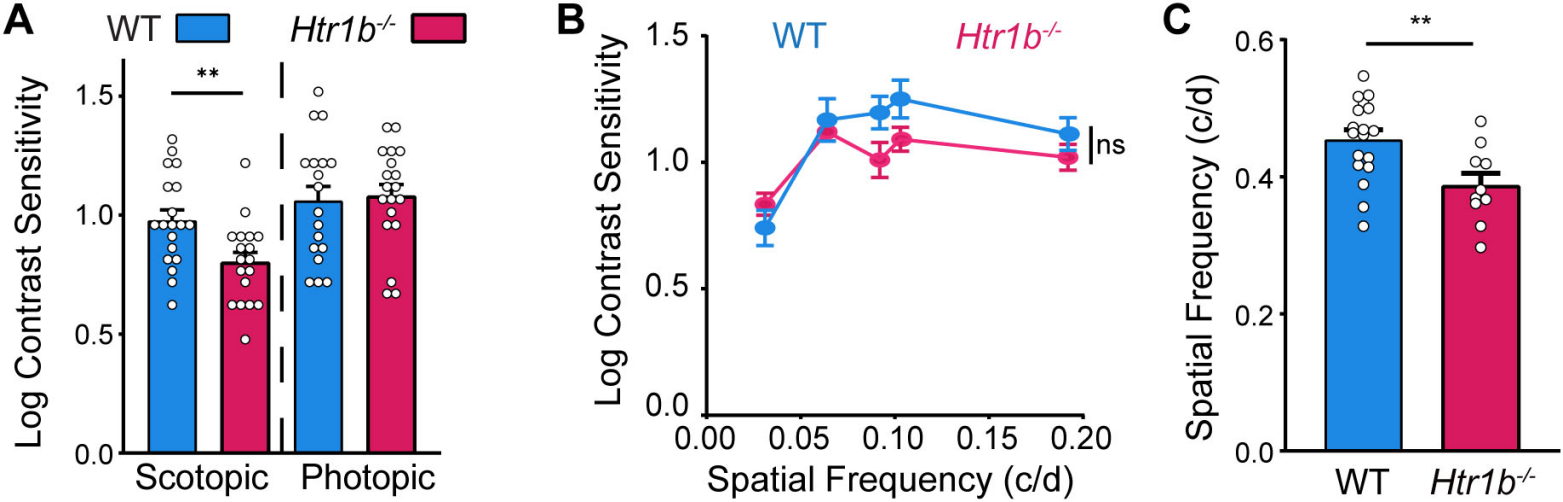
*Htr1b*^–/–^ mice have aberrant visual function **(A)** Scotopic contrast sensitivity was significantly reduced in *Htr1b*^–/–^ mice compared to WT control mice, whereas photopic contrast sensitivity was not affected (*Htr1b*^–/–^ *N* = 10 mice; WT *N* = 10 mice.; **P < 0.01). **(B)** Photopic contrast sensitivity function plotted over 5 spatial frequencies (c/d = cycles/degree) was not different between *Htr1b*^–/–^ and WT mice (*Htr1b*^–/–^ *N* = 5 mice; WT *N* = 9 mice). **(C)** *Htr1b*^–/–^ mice had significantly reduced photopic spatial frequency thresholds compared to WT mice (*Htr1b*^–/–^ *N* = 5 mice; WT *N* = 9 mice; **P < 0.01).

Next, we determined the effects of *Htr1b* loss of function on visual function. To do so, we used the optokinetic response (OKR) to assess contrast sensitivity at peak spatiotemporal frequency under photopic and scotopic conditions (Cowan et al., 2016a; van der Heijden et al., 2016). We found that scotopic contrast sensitivity was significantly reduced in *Htr1b*^–/–^ mice when compared with control mice (Figure 4A; **P < 0.01). However, photopic contrast sensitivity was unaffected (*P* > 0.05). To investigate possible subtle effects on photopic contrast sensitivity, we generated a contrast sensitivity function over 5 spatial frequencies. We found that contrast sensitivity was not affected at any spatial frequency in *Htr1b*^–/–^ mice (Figure 4B; *P* > 0.05 at all spatial frequencies). Finally, we measured photopic spatial acuity thresholds (visual acuity) and found that they were significantly reduced in *Htr1b*^–/–^ mice compared to controls (Figure 4C; **P < 0.01). These results suggest that *Htr1b* function is necessary for several aspects of visual processing.

### 3.4 *Htr1b*^–/–^ animals have altered retinal electrical responses *in vivo*

**FIGURE 5:**
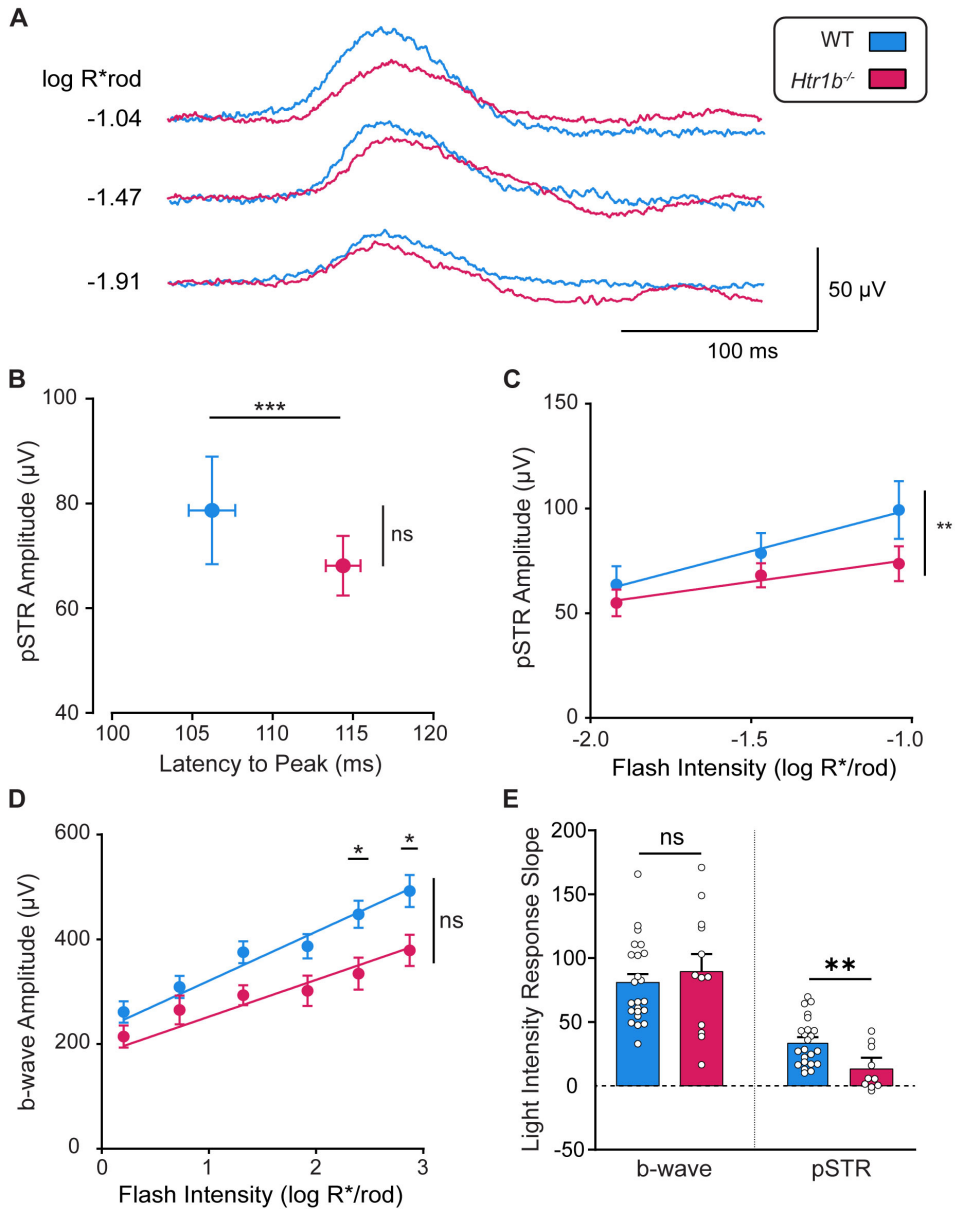
*Htr1b*^–/–^ mice have altered retinal electrophysiology. **(A)** Representative pSTR waves were obtained at three light intensities ranging from –1.92 to –1.04 log photoisomerizations/rod. **(B)** pSTR amplitude and the latency to peak amplitude were determined at a light intensity of –1.04 log R*/rod. The latency to peak amplitude is increased (delayed) in *Htr1b*^–/–^ mice compared to WT mice (***P < 0.001). However, there was no significant difference in peak pSTR amplitude. **(C)** pSTR peak amplitudes are plotted over three light intensities. There is no significant difference in peak amplitude at any light intensity. **(D)** B-wave peak amplitudes are plotted over 6 light intensities from 0.20 to 2.87 log R*/rod. These are reduced in *Htr1b*^–/–^ mice at only the brightest intensities. **(E)** The light intensity response slope was calculated as the linear regression slope of the b-wave or pSTR amplitude (μV) over increasing light intensities (Shen et al., 2018). The pSTR but not b-wave light intensity slope is significantly decreased in *Htr1b*^–/–^ mice compared to WT mice (**P < 0.01). *Htr1b*^–/–^ *N* = 8 mice; WT *N* = 13 mice.

Next, we evaluated the consequences of *Htr1b* loss of function *in vivo* using the electroretinogram (ERG). We found that the positive scotopic threshold response (pSTR), which reflects the summed electrical response of RGCs (Saszik et al., 2002), showed a normal amplitude but significantly delayed time latency in *Htr1b*^–/–^ mice compared to WT mice (Figures 5A, B; ***P < 0.001). While the pSTR amplitude was not impacted at any individual light intensity, an attenuated slope trend was present (Figure 5C; **P < 0.01). The amplitude of the b-wave, which reflects bipolar cell responses, revealed a decrease in amplitude at only the highest light intensities (Figure 5D; *P < 0.05; 2.4, 2.9 log R*/rod), but no differences in latency (not shown). The a-wave, which reflects photoreceptor responses, showed no significant differences (not shown). We also found that the pSTR but not the b-wave of *Htr1b*^–/–^ mice showed reduced sensitivity in response to increasing light stimuli compared to WT controls (Figure 5E; **P < 0.01). Overall, these findings suggest that loss of *Htr1b* function affects RGC physiology, specifically temporal processing and light adaptation responses.

### 3.5 *Htr1b*^–/–^ animals have altered RGC responses and sensitivity to 5-HT

Since we detected a strong ERG phenotype in the RGCs of *Htr1b*^–/–^ mice, we extended this analysis to individual RGCs using *ex vivo* multielectrode array (MEA) recordings. We recorded light-stimulated RGC spikes from both WT and *Htr1b*^–/–^ retinas under baseline conditions and after adding 100 μm 5-HT to the bath solution (see Methods).

We first identified RGCs that showed a change in action potential firing rate when 5-HT was added to the bath solution to identify serotonin-responsive neurons (Table 4). Most cells responded to 5-HT, while a minority did not. Interestingly, removing *Htr1b* preferentially increased the responsiveness of OFF but not ON RGCs to 5-HT compared to WT controls (*P < 0.05; Chi-Square). We excluded RGCs that showed no response to 5-HT from further analysis as they likely either lacked 5-HT receptors or were not regulated by 5-HT via retinal circuitry. ON-OFF cells were also excluded from further analysis due to their low sample size in our recordings.

**TABLE 4:** Summarized RGC responses to 5-HT.

	ON		OFF		ON-OFF	
	Response	No response	Response	No response	Response	No response
WT	26 (68%)	12 (32%)	20 (56%)	16 (44%)	8 (80%)	2 (20%)
*Htr1b* ^–/–^	52 (73%)	19 (27%)	46 (79%)	12 (21%)	10 (63%)	6 (37%)

Summarized responses and percentage distribution of WT and *Htr1b*^–/–^ RGCs to the application of 5-HT, indicating whether RGC activity changed or not. A greater proportion of *Htr1b*^–/–^ OFF RGCs responded to 5-HT compared to WT OFF RGCs (*P < 0.014; Chi-square), while ON RGCs did not show significantly different response rates between the two genotypes (*P* = 0.60). *N* = 8 WT retinas; 6 *Htr1b*^–/–^ retinas.

**FIGURE 6:**
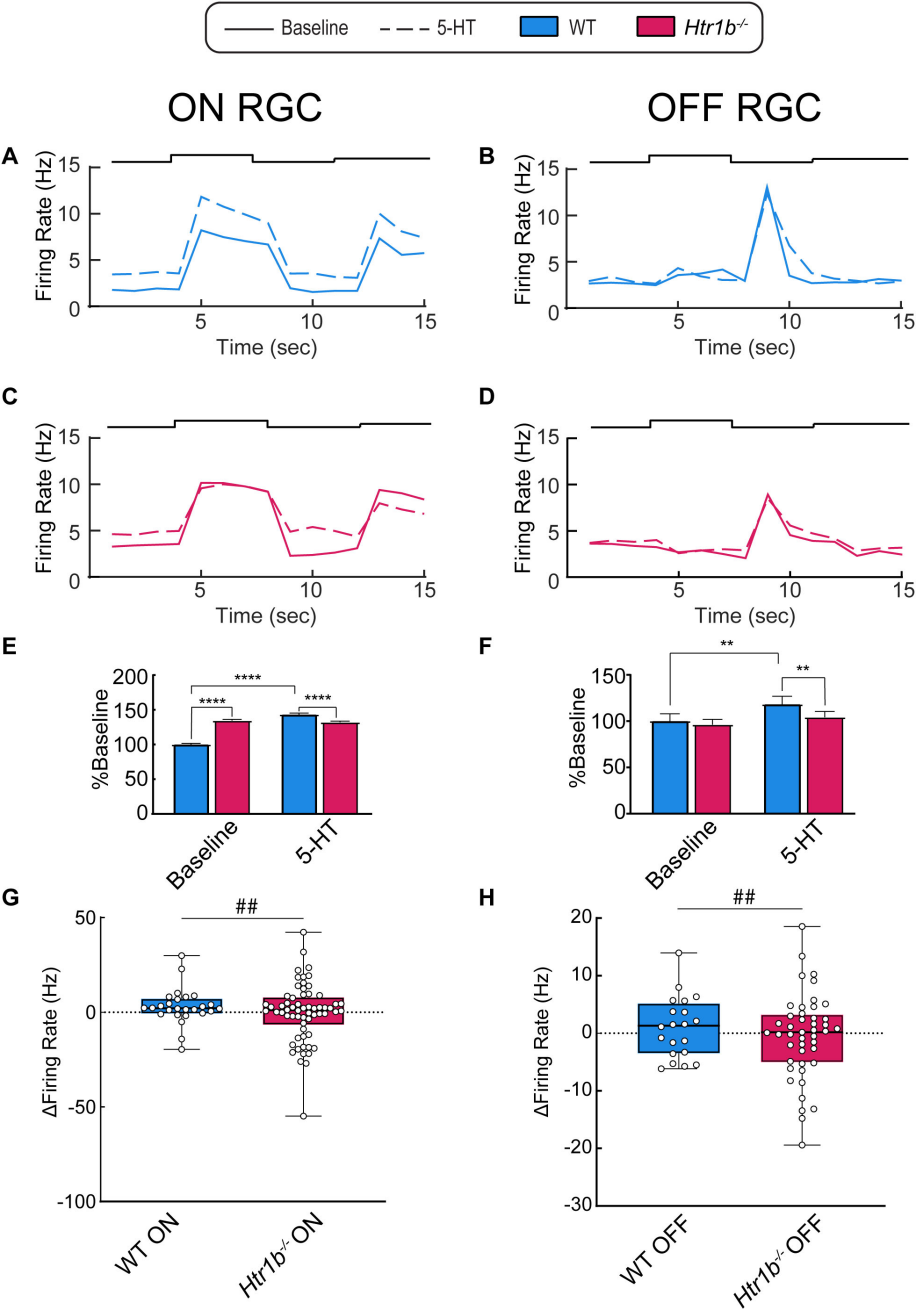
*Htr1b*^–/–^ RGCs have altered RGC responses and sensitivity to 5-HT. **(A-D)** Averaged firing rate of RGCs from WT (blue) and *Htr1b*^–/–^ (red) RGCs at baseline (solid line) and after bath application of 100 μM 5-HT (dash). **(E,F)** The average evoked firing rates in ON and OFF RGCs, normalized to baseline wildtype conditions. **(E)** ON *Htr1b*^–/–^ RGCs have increased baseline activity relative to WT controls (****P < 0.0001; 2-way ANOVA) but an unchanged response to 5-HT. **(F)** OFF *Htr1b*^–/–^ RGCs have normal baseline activity but a blunted response to 5-HT relative to WT controls (**P < 0.01; 2-way ANOVA). **(G,H)** Individual ON **(G)** and OFF **(H)** RGC firing rates in response to 5-HT. The relative change in firing rate is indicated for each RGC. 5-HT did not significantly increase the median firing rate (thick black bar) of either ON or OFF RGCs from either WT or *Htr1b*^–/–^ retinas. However, exclusively in *Htr1b*^–/–^ retinas, 5-HT strongly increased the variability of RGC responses (F-test: ^##^*P* < 0.01). **P < 0.01; ****P < 0.0001; N *Htr1b*^–/–^ = 52 ON, 46 OFF cells across 6 retinas; N WT = 26 ON, 20 OFF across 4 retinas.

The average firing rate across all recorded WT and *Htr1b*^–/–^ cells is shown in Figures 6A–D. WT ON RGCs showed a peak firing rate increase in response to 5-HT application (Figure 6A), while *Htr1b*^–/–^ ON RGCs did not (Figure 6C). In OFF RGCs, peak firing rate did not change after 5-HT application regardless of genotype (Figures 6B, D). To better compare WT and *Htr1b*^–/–^ RGCs, we quantified the evoked firing rates of the RGCs by analyzing the evoked firing rate during the light stimulus step (5–8 s for ON step; 9–12 s for OFF step) and normalized all firing rates to baseline WT RGCs to better understand relative changes in magnitude dynamics (Figures 6E, F). When normalized to WT baseline peak evoked firing rates, ON and OFF RGCs responded differently. In ON RGCs, WT RGCs showed an increase in firing with 5-HT (****P < 0.0001). In contrast, *Htr1b*^–/–^ ON RGCs did not show an increase in firing with 5-HT but did show increased baseline firing rates compared to WT ON RGCs (****P < 0.0001). In OFF RGCs, WT cells showed an increase in evoked firing with 5-HT (**P < 0.01), but this is due to prolonged firing after the light stimulus as opposed to an increased peak firing magnitude (Figures 6B, F). In contrast to *Htr1b*^–/–^ ON RGCs, *Htr1b*^–/–^ OFF RGCs had similar baseline evoked firing rates to WT cells but a blunted response to 5-HT (**P < 0.01).

To further explore the effects of 5-HT on firing rates, we tracked changes in the firing rates of individual RGCs after 5-HT application (Figures 6G, H). Strikingly, we found that both *Htr1b*^–/–^ ON and OFF RGCs showed significantly more variability in response to 5-HT when compared to WT controls (^##^*P* < 0.01; F-test). This suggests that *Htr1b* plays a role in stabilizing the magnitude of RGC responses. Together, these findings suggest that *Htr1b* is necessary in ON RGCs to prevent hyperactive basal responses and in OFF RGCs to achieve the full evoked, excitatory effect of 5-HT. Absence of *Htr1b* also resulted in an increased proportion of OFF RGCs responding to 5-HT. Finally, removal of *Htr1b* resulted in more variability in firing rates in both ON and OFF RGCs response to 5-HT.

## 4 Discussion

In this study, we performed seminal experiments on *Htr1b* expression and function in the retina. We localized *Htr1b* transcript and HTR1B protein to RGCs and the inner retina. We found that *Htr1b*^–/–^ mice are grossly normal anatomically, yet exhibit defects in inner retinal electrical activity, RGC function, and vision. Interestingly, our MEA data suggests that *Htr1b* may act differently in ON and OFF RGCs. These novel phenotypes have several implications for 5-HT function in retinal biology.

5-HT plays an important role in the central nervous system by regulating neuronal proliferation, differentiation, migration, and survival as well as synapse formation, stabilization, and plasticity (Gaspar et al., 2003; Bonnin and Levitt, 2011; Sitko et al., 2018). The role of 5-HT in retinal development is more ambiguous, but 5-HT has been shown to impact retinal development by regulating neurite outgrowth and refinement of RGC axons (Upton et al., 1999; Trakhtenberg et al., 2017). In the brain, *Htr1b*^–/–^ mice do not exhibit overt developmental abnormalities (Saudou et al., 1994; Ramboz et al., 1996). Consistent with these findings, our data show that *Htr1b* loss does not grossly disrupt retinal anatomy. However, these experiments were limited by low resolution experiments which may not detect more subtle phenotypes.

Our data show that *Htr1b* loss of function had a preferential effect on contrast sensitivity in scotopic rather than photopic vision. This suggests that *Htr1b* may play a crucial role in detecting visual signals in low-light conditions. ERG analysis showed that the pSTR, which reflects bulk RGC activity, showed significant temporal processing delays under low light conditions. The b-wave, which reflects bipolar cell activity, showed reduced amplitudes. These experiments, together, suggest that *Htr1b* works preferentially in the inner retina. Previous studies have shown that 5-HT can affect gain modulation in the scotopic pathways by acting through several retinal cells including RGCs, amacrine cells, and rod bipolar cells (Brunken and Daw, 1988a; Daw et al., 1990), and these processes may be mediated by *Htr1b*.

The effects of adding 5-HT to a neuronal bath on firing rate can be complex and depend on the specific neuron being studied and concentration of 5-HT. Consistent with other studies, we found that adding 5-HT to the bath led to a largely excitatory effect to the neuron firing rates (Rav-Acha et al., 2008; Huang et al., 2017). This suggests that under normal conditions, 5-HT enhances neuronal excitability.

Our findings also showed that *Htr1b* loss led to increased basal firing in ON RGCs, suggesting that *Htr1b* normally acts to suppress neuronal excitability. HTR1B is a well-characterized Gi/o-coupled receptor. When activated, the βγ subunit of the G-protein dissociates and binds to GIRK channels, leading to membrane hyperpolarization. Consequently, the net effect of 5-HT on RGCs likely reflects a balance between inhibitory and excitatory receptor signaling. *Htr1b* disruption may perturb this equilibrium, resulting in increased membrane excitability. In contrast, OFF RGCs exhibited normal firing baseline firing in *Htr1b* mutants but showed a blunted response to 5-HT, suggesting *Htr1b* may play a disinhibitory role, which allows other receptors to amplify serotonin-mediated firing rates. This potential new mechanism will require investigation of other 5-HT receptors to confirm.

The disparate responses of ON and OFF RGCs in the absence of *Htr1b* could also stem from differences in 5-HT receptor expression levels and patterns (Figure 1E). One explanation involves the *Htr1d* gene, which shares similar homology and function to *Htr1b*, can form heterodimers with it, and therefore may be able to compensate for the lack of *Htr1b* function (Xie et al., 1999; Mitroshina et al., 2023). *Htr1d* is expressed at higher levels in ON compared to OFF RGCs and therefore may show stronger compensatory effects in ON cells in the absence of *Htr1b*. Alternatively, *Htr1b* and *Htr1d* may exert distinct or overlapping influences on RGC responses to 5-HT.

Another explanation is that ON and OFF RGC activity is maintained through distinct mechanisms (Margolis and Detwiler, 2007; Zhang et al., 2016). ON cell activity is maintained by upstream synaptic input while OFF cell activity is more heavily modulated by intrinsic properties. Thus, *Htr1b* phenotypes in ON RGCs may be related to abnormal synaptic input whereas phenotypes in OFF RGCs may be related to dysfunctional intrinsic activity.

Removal of *Htr1b* also increased variability in serotonin-dependent firing rates, which could arise via any of the mechanisms above. HTR1B’s downstream effect is to activate hyperpolarizing currents via GIRK channels which play a stabilizing role in maintaining neuron firing regularity (Luo et al., 2022). Inhibition of potassium channels has been linked to destabilized firing rates by increasing firing irregularity (Iyer et al., 2017) and increasing sensitivity to cocaine (McCall et al., 2019). Future studies will more deeply investigate the role of GIRK/HTR1B in cell-firing dynamics.

Finally, removal of *Htr1b* led to an increased proportion of OFF RGCs that responded to 5-HT compared to WT OFF RGCs. This suggests that *Htr1b* is not the primary receptor that responds to 5-HT. Instead, *Htr1b* works in a modulatory fashion alongside other 5-HT receptors, shaping their activity.

Our study has several limitations. We used a germline KO line, which removes gene function from all cells and precludes distinguishing cell-autonomous and non-cell-autonomous effects. Future experiments that use conditional KO lines or receptor-specific pharmacology will be better able to parse out the cell-specific contribution of *Htr1b* to the pSTR and RGC firing rates. Such experiments can also allow targeted assessment of αRGCs. Additionally, because 12 *Htr* genes are expressed in RGCs, compensatory upregulation of other 5-HT receptors may occur following *Htr1b* loss. Future work examining these compensatory mechanisms will help clarify regulatory networks among co- and differentially expressed *Htrs*.

In summary, we found that *Htr1b* is an important receptor in retinal serotonergic circuitry, and can modulate inner retinal electrical activity, RGC responses, and visual behavior. Uncovering additional details about the cell-specific contribution of *Htr1b*, the molecular mechanism underlying *Htr1b* activity, the distinct effects in ON vs OFF RGCs, and the role of other 5-HT receptors in serotonergic function will be vital to discovering potential serotonin-derived interventions for ophthalmic diseases and fully understanding the role of 5-HT in retinal and RGC function.

## Significance statement

Our results show that loss of *Htr1b* function results in abnormal retinal physiology and visual function despite normal retinal anatomy. These experiments suggest that *Htr1b* plays an important and previously unrecognized role in 5-HT action in the retina.

## Data Availability

The datasets presented in this study can be found in online repositories. The names of the repository/repositories and accession number(s) can be found below: https://doi.org/10.5061/dryad.bk3j9kdps.
